# Role of optical imaging in oral cancer and oral potentially malignant disorders: implications for oral submucous fibrosis

**DOI:** 10.3389/froh.2025.1760778

**Published:** 2026-01-13

**Authors:** Surendra Kumar Acharya, Nurul Izyan Zainuddin, Yee Fan Choon, Mamata Rai, Tania Saskianti, Wanninayake Mudiyanselage Tilakaratne, Vui King Vincent-Chong

**Affiliations:** 1 Department of Oral and Maxillofacial Clinical Sciences, Faculty of Dentistry, University of Malaya, Kuala Lumpur, Malaysia; 2 Department of Oral and Maxillofacial Surgical Sciences, Faculty of Dentistry, MAHSA University, Jenjarom, Selangor, Malaysia; 3 Department of Nursing, Manmohan Memorial Institute of Health Sciences, Tribhuvan University, Kathmandu, Nepal; 4 Department of Pediatric Dentistry, Faculty of Dental Medicine, Universitas Airlangga, Surabaya, Indonesia; 5 Department of Oral Oncology, Roswell Park Comprehensive Cancer Center, Buffalo, NY, United States

**Keywords:** artificial intelligence (AI), betel quid, non-invasive optical imaging, oral submucous fibrosis, potentially malignant disorder

## Abstract

Oral submucous fibrosis (OSF) is a chronic, potentially malignant disorder characterized by progressive stromal fibrosis and epithelial atrophy, leading to functional loss and an increased risk of malignant transformation. Areca nut consumption remains the principal etiological factor in South and Southeast Asia. Despite its distinct clinicopathological features, OSF assessment relies largely on clinical examination and invasive biopsy, underscoring the need for non-invasive approaches capable of interrogating tissue structure and composition. Optical imaging (OI) technologies, including confocal-based imaging, optical coherence tomography (OCT), narrow-band imaging (NBI), and Raman spectroscopy (RS), have been widely investigated in oral potentially malignant disorders (OPMDs) and oral squamous cell carcinoma (OSCC). However, the applicability of OI to OSF remains undefined. The aims of this narrative review are to: 1) critically synthesize current OI evidence in oral mucosal disease, and 2) evaluates the biological plausibility and technical limitation of applying these modalities to OSF from a pathobiology point of view. Highlighted in this review are the cellular and extracellular matrix alterations that may generate measurable optical signals and the paucity of OSF-specific validation studies, in addition to discussing constraints related to imaging penetration depth and disease grading, and outlining future research directions, including extracellular-matrix-focus optical biomarkers and artificial intelligence-assisted analysis. Collectively, this work positions OI in OSF as a hypothesis-generating and exploratory domain requiring rigorous, pathology-correlated investigation before clinical translation.

## 1 Introduction

Approximately 600 million individuals worldwide are estimated to habitually chew areca nut, making it one of the most commonly used psychoactive substances globally. A habit highly prevalent in South Asia, parts of Southeast Asia, aand the Pacific Island, areca nut is most frequently consumed as a principal component of betel quid (BQ), prepared typically with areca nut wrapped in a leaf of Piper betle, slaked lime, catechu, spices, and often tobacco. In India, these quids are commercially available products such as Pan Masala and Gutka and particularly popular when combined with tobacco. The prevalence of BQ varied considerably across populations, raning from 0.8% to 46.3%. Arecoline, a major alkaloid found in areca nut has been classified as a Group 2B carcinogen by the International Agency for Research on Cancer (IARC) in 2021, indicating its potential carcinogenicity to humans. Arecoline exposure induces a range of pathological mucosal alterations in animal models, including epithelial hyperplasia, dysplasia, papilloma formation, and progression to invasive squamous cell carcinoma (SCC) preclinically (1–3). In the human oral cavity, the use of areca nut is an etiological factor for oral submucous fibrosis (OSF), a potentially malignant disorder (PMD) (4–6). OSF is defined as “a chronic, insidious disease that affects the oral mucosa, initially resulting in the loss of fibro elasticity of the lamina propria and, as the disease advances, results in fibrosis of the lamina propria and the submucosa of the oral cavity along with epithelial atrophy” (7). Clinically, the affected oral mucosa may appear persistently white, blanched, and marble-like with tough, leathery texture and palpable whitish fibrous bands. Severity of mouth opening limitation, the coexistence of other OPMDs, and ultimately, the onset of oral squamous cell carcinoma (OSCC) constitutes the functional grading of OSF clinically (8, 9). Globally, the prevalence of OSF was 4.96% (95% CI = 2.28–8.62) (10). An overall malignant transformation rate of 4.2%–6% has been reported (11–13). Recently, the International Agency for Research on Cancer (IARC) has estimated a 77% increase in the global burden of cancer in 2050 (14). 258,440 cases of lip and oral cavity cancer cases with a 75.1% mortality rate had been recorded in the continent of Asia, where BQ use is endemic (15, 16). The diagnosis of OSF is based on its clinical presentation and histopathology. Currently, conventional oral examination (COE) is the recommended, best first-line approach in the early detection of OPMDs and OSCC (17, 18), however, the diagnostic accuracy of the method warrants further improvement (19, 20), given the subjectivity of the method and wide spectrum of signs and symptoms of OSF (21, 22). Tissue biopsy has been the gold standard in diagnosing and grading OSF (9, 23); however, none of the currently used diagnostic/staging systems can identify the high-risk population or predict malignant transformation accurately (8). Hence, other adjuvant methods have been studied to improve the accuracy of detection and staging systems (24, 25). Several non-invasive tools have been explored to improve the sensitivity and specificity of COE and aid in the biopsy and diagnosis of OPMDs and OSCC (26–28). These adjuvant methods include 1) vital dyes (toluidine blue, Lugol's iodine, methylene blue, and rose Bengal), 2) oral cytology (oral exfoliative biopsy/brush biopsy, and fine needle aspiration cytology), 3) light-based detection systems (chemiluminescence techniques such as ViziLite, Vizilite Plus, Microlux/DL, Orascoptic DK, and autofluorescence techniques such as VELscope), and 4) optical imaging and spectroscopic techniques (29–34). Optical imaging (OI) and spectroscopy represent the latest advancement in the effort to develop non-invasive and affordable methods of early cancer detection and screening (35, 36). Reflectance Confocal Microscopy (CRM), Optical Coherence Tomography (OCT), Raman Spectroscopy (RS), and Narrow Band Imaging (NBI) have demonstrated their ability to detect subtle structural and biochemical changes in epithelial-predominant OPMDs in real time and non-invasively (37). Importantly, the majority of these data are derived from epithelial-predominant disorders such as leukoplakia and OSCC, and cannot be directly extrapolated to OSF, a condition fundamentally driven by progressive stromal fibrosis. Hence, OI techniques have been proposed as potential adjunctive tools for non-invasive assessment of oral mucosal disease, particularly in the context of OPMDs and OSCC, although their applicability to OSF remains insufficiently validated (38). As outlined above, OSF is characterized by distinct epithelial, stromal, and extracellular matrix alterations that are biologically capable of generating measurable optical signals. However, whether current OI and spectroscopic techniques can adequately interrogate the depth and extent of OSF-related fibrosis remains insufficiently validated. Accordingly, the intent of this review is threefold: (1) to delineate the cellular, stromal, and extracellular matrix alterations characteristic of OSF as the biological substrate for optical interrogation; (2) to critically synthesize existing OI evidence derived primarily from OPMDs and OSCC, explicitly highlighting the paucity of OSF-specific validation studies; and (3) to evaluate, from a pathobiology-led perspective, the technical feasibility, limitations, and biologically plausible future research directions, including high-resolution digital biopsy, artificial intelligence-assisted interpretation, and standardized image annotation frameworks, required to responsibly advance OI research in OSF.

## 2 Materials and methods

Databases, including PubMed, Google Scholar, Semantic Scholar, and Science Direct, were searched from 1996 to 2025 using various combinations of the following keywords: “oral submucous fibrosis”, “oral premalignant disorders”, “collagen fibers”, “histology”, “ultrastructural”, “malignant transformation,” “areca nut”, “non-invasive biopsy”, “optical imaging”, “optical biopsy”, “adjuvant tools”, “Artifical intelligence”, “Optical Coherence Tomography (OCT)”, “Raman Spectroscopy (RS)”, “Narrow Band Imaging (NBI)” and, “Confocal Microscopy (CM)”. Original experimental studies, reviews, editorial letters, book chapters, opinions, and abstracts published in English considering optical imaging of OSF were considered and included in this narrative review.

## 3 Ultrastructural and light microscopic alterations of OSF

Ultrastructural and light microscopic alterations in the epithelium and connective tissue of OSF are presented in this section to establish the cellular and extracellular matrix-based rationale for considering optical imaging and spectroscopic interrogation. These well-characterized pathological changes provide the biological context for understanding how disease-related alterations in tissue architecture, composition, and organization may generate measurable optical signals.

### 3.1 Fibrotic alterations of the connective tissue matrix

One of the hallmarks of OSF is the progressive deposition of excessive collagen as the disease advances. Since its first report in 1966 (39, 40). Studies have been conducted to examine the properties of these collagen fibers, findings of which are presented in this section as the rationale for the application of OI techniques in OSF.

Previous ultrastructural studies have demonstrated notable differences in the connective tissue architecture between normal oral mucosa and OSF. Using scanning electron microscopy (SEM), Binnie and Cawson observed that collagen fibrils in normal connective tissue appeared as uniformly sized, well-organized, and densely packed bundles embedded within a ground substance matrix. However, fibrillar organization was markedly disrupted in the connective tissue from OSF specimens, with no true bundle formation. Collagen fibers were not irregularly dispersed within the ground substance in OSF, but were condensed at approximately 210–240 fibrils/*μ*m^2^, as compared to 170–180 fibrils/μm^2^ in normal tissue. Furthermore, there was a significant reduction in the mean diameter of collagen fibrils (2.35 μm) in OSF than that of normal tissue (3.23 μm). Additionally, an increased presence of reticulin, elastin, and oxytalan, indicative of complex and aberrant remodelling of OSF extracellular matrix was also reported. Fibrotic progression in OSF is accompanied by both quantitative and qualitative alterations in connective tissue components, which maybe responsible for the aforementioned functional impairment and disease progression observed clinically (41). Van Wyk and colleagues further reported that the thinner fibers were Type III collagen fibrils and concentrated adjacent to the basement membrane, while thick Type I collagen bundles were noted deeper in the lamina propria of moderate and advanced OSF than in controls (42). In addition, Reichart et al. showed marked deposition of amorphous material next to the collagen fibrils in the subepithelial connective tissue of OSF (43). The biological property of the amorphous material was, however, undetermined (42). Morphological alterations in OSF were well documented in a SEM study. SEM analysis revealed significant degeneration and fragmentation of the connective tissue cores in OSF, with structural disruptions becoming more pronounced in correlation with disease progression. One of the hallmark features observed was the progressive flattening of the epithelial-connective tissue interface, indicative of advanced architectural remodeling. In agreement with earlier studies, the distribution of elastic fibers also demonstrated notable changes. Elastic fibers which appeared as fine, uniformly distributed strands in healthy oral mucosa were thickened and aggregated into dense bundles, particularly within the deeper subepithelial regions of connective tissue in OSF-affected tissue. Hence, areca nut consumption cumulatively impacts connective tissue integrity and extracellular matrix of healthy oral mucosa, contributing to the characteristic stiffness and loss of mucosal pliability associated with the disease (44).

Light microscopic studies further corroborated the above findings (45). Specialized histochemical stains had enabled the identification of collagent types I and III, as well as elastic fibers, under light and polarized microscopy in the histological evaluation of normal oral mucosa and OSF. Notable difference in the orientation and density was observed in OSF specimens despite their presence in both normal and OSF tissues. Collagen and elastic fiber were more randomly and haphazardly arranged in healthy mucosa. In advancing stages of OSF, however, a reorganization and marked increase in the density of type 1 collagen fibers with a parallel orientation relative to the overlying epithelium was observed. However, a reduction in the density of collagen fibers types I and III was noted in advanced OSF, suggesting a stage-dependent remodeling of the extracellular matrix in OSF, with increased fibrosis and loss of matrix heterogeneity contributing to the clinical progression of the disease (46–49). Such collagen fiber thickness and orientation alterations have been documented in both OSF and OSCC. Arora et al. demonstrated distinct difference in the degree of collagen birefriengence among normal mucosa, OSF, and OSCC tissues using Picosirius Red staining under polarized light microscopy. Consistently, markedly thick and densely packed collagen fibers were observed in OSF specimens, in contrast with the presence of thin, loosely arranged collagen fibers, appearing as green-yellow birefringence in OSCC samples. These findings reiterate progressive remodeling and degradation of the extracellular matrix architecture during malignant transformation, with potential implications for tissue stiffness, invasiveness, and diagnostic assessment (50, 51). Investigation with Masson's trichrome (MT) and Verhoeff-Van Gieson (VVG) special staining also demonstrated similar changes, where collagen fibers were arranged in thick bundles in the deeper part of the lamina propria in OSF and thin, fragmented bundles in OSCC. Such differences were also noted for elastic fibers in the study (52). A study by Utsunomiya et al. examined how extracellular matrix (ECM) components change across different stages of OSF. Furthermore, ECM remodeling that contributes to OSF progression and functional impairment, with specific molecules potentially serving as markers for disease was demonstrated with immunohistochemistry and *in situ* hybridization studies on 40 OSF tissue samples showing increased expression of tenascin, perlecan, fibronection, and collagen type III in early-stage OSF. In intermediate stages, elastic and collagen type I accumulated around muscle fibers, and in advanced OSF, ECM diversity diminished, and collagen type I dominated, extending into muscle tissue (53). In summary, observable microscopic alterations in the thickness and orientation of collagen and elastic fibers have been documented as OSF develops clinically from its early to advanced stages and finally into overt OSCC (54–56).

### 3.2 Epithelial alterations

In addition to changes in the connective tissue, a higher malignant transformation rate has been reported in OSF with epithelial alterations. OSF with oral epithelial dysplasia (OED) was 1.89 times more likely to transform into OSCC and exhibited the highest malignant transformation rate among other epithelial alterations, such as verrucous hyperplasia, hyperkeratosis, or epithelial hyperplasia (57–59). Regardless of other risk factors, OED has emerged as an independent risk factor for malignant transformation (60, 61). OED grading is the gold standard in the histopathological assessment of malignant transformation risk in oral epithelium. The system is however inherently subjective and arbitrary. Even though the system has been revised to enhance its predictive accuracy, its diagnostic reliability and clinical utility are continuously being improved through standardization and objective criteria are needed further (62, 63). Though epithelial atrophy and dysplastic alterations may influence epithelial thickness measurement, cellular morphology, and epithelial-stromal interface characteristics, which are parameters accessible to high-resolution OI modalities, the aforementioned subjectivity inherent in conventional histopathological grading further underscores the need for objective, non-invasive correlates in the applicability of OI in OSF.

### 3.3 Blood vessel alterations

#### 3.3.1 Early stage

Vasodilation and congestion frequently observed in this stage indicates an inflammatory reaction to areca nut components (64).

#### 3.3.2 Intermediate stage

Vessel constriction observed in this stage, as fibrosis advances, results in reduced luminal diameter and compromised blood flow as the proliferating collagen fibres exert pressure on blood vessels, leading to their constriction (65).

#### 3.3.3 Advanced stage

In advanced stage, obliteration or narrowing of blood vessels can be brought about by extensive fibrosis and hyalinization, leading to significant tissue ischemia (66). Epithelial thickness, blood vessel area, diameter, and perimeter did not change consistently in association with grade in a morphometric assessment of histological sections of OSF (64). Sirsat et al. and Fang et al. suggested that dilatation and congestion of blood vessels occurred in the early stages and constriction to obliteration at the later stages (67). Mean vascular dilatation occurred because of an adaptive response to compensate for tissue ischemia/hypoxia. Histopathological changes in the epidermis and blood vessels remain subjective (68).

**Figure 1:**
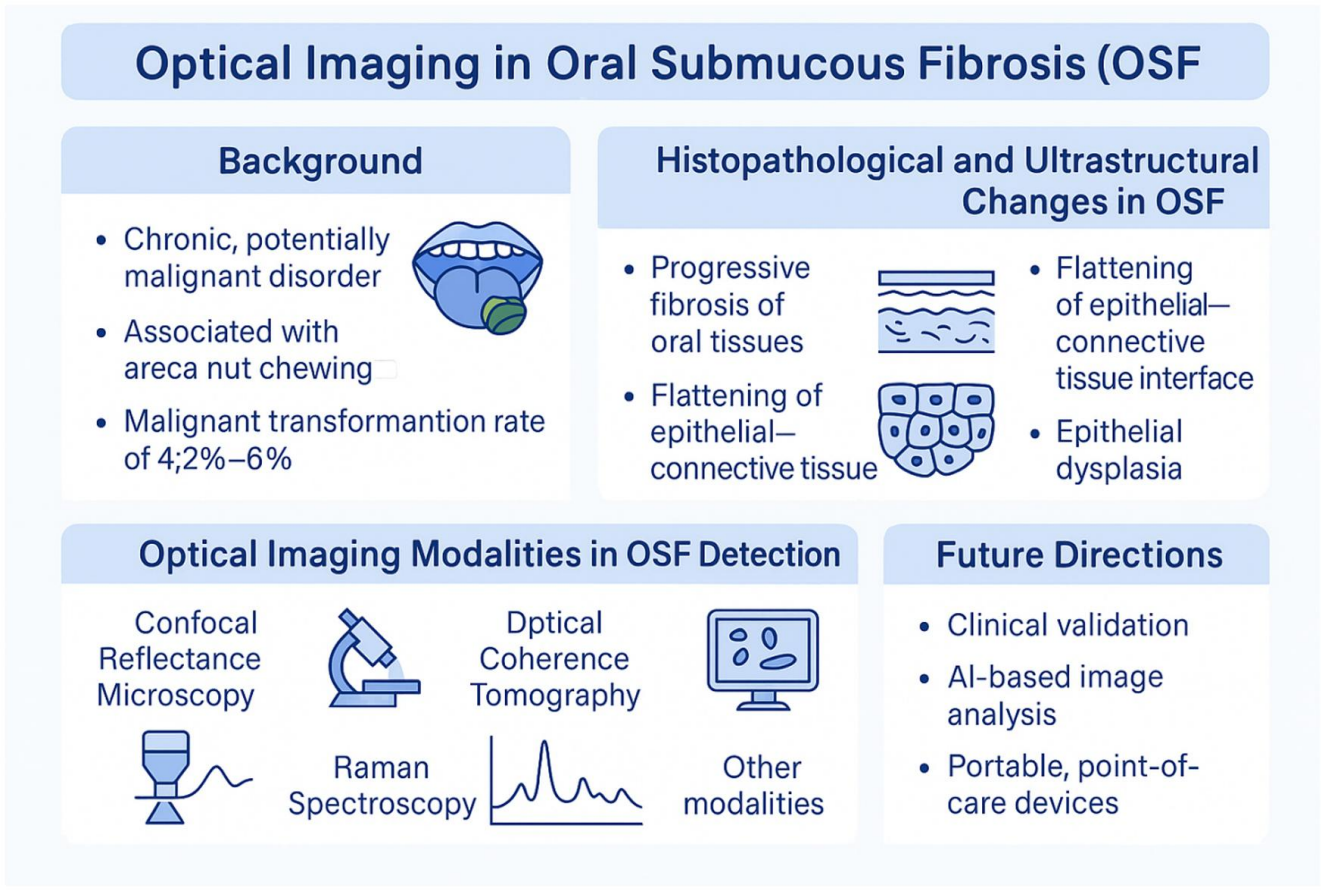
Pathobiology-led rationale for optical imaging in oral submucous fibrosis. Progressive extracellular matrix modelling, epithelia atrophy, and vascular alteration in OSF generate biologically plausible optical signatures. While optical imaging modalities are validated in OPMDs and oral squamous cell carcinoma, OSF-specifi evidence remains limited. This review highlights key biological targets, technical constraints, and future directions for pathology-correlated validation.

These vascular changes contribute to the clinical observation of mucosal stiffness, trismus, and a heightened risk of malignancy in OSF. Understanding these alterations is crucial for developing targeted therapeutic strategies aimed at improving vascular health and mitigating disease progression in OSF patients. Extravasated inflammatory cells may be noted surrounding the blood vessels, and the collagen fibres exhibit peri-vascular cuffing. The blood vessels tend to get compressed due to the hyalinization of the surrounding collagen fibres (69). With further progression of fibrosis and hyalinization, a decrease in the density of micro vessels occurs due to diminished levels of mediators of angiogenesis (70). With these vascular alterations contribute to OSF pathophysiology and clinical manifestations, they represent secondary changes derived by progressive fibrosis. Consequently, optical techniques targeting vascular patterns are more likely to provide indirect or supportive information rather than direct measurement of fibrotic severity. Together, these structural, epithelial, and vascular alterations define the pathological landscape of OSF and form the biological basis upon which the potential and limitations of OI and spectroscopic modalities must be evaluated (Figure 1).

## 4 Optical biopsy in OPMDs/OSCC

Optical biopsy is defined as *in situ* imaging of tissue microstructure with a resolution approaching that of histology, but without the need for tissue excision and processing (71). Based on conclusions from systematic reviews, this section focuses its discussion on con-focal reflectance microscopy (CRM) (72), optical coherence tomography (OCT) (73), narrow-band imaging (NBI) (26), and Raman spectroscopy (RS) (74) as potential optical biopsy techniques with established evidence in OPMDs/OSCC and potential, yet insufficiently validated, relevance to OSF. In keeping with the aim of this review, we distinguish between 1) validated applications in epithelial-dominant lesions, and 2) biologically plausible., ECM-anchored hypotheses for OSF that require pathology-correlated studies, including a discussion on the increasing integration of AI in the interpretation of images captured by these optical techniques (Table 1).

**Table 1:** Optical imaging modalities in oral mucosal disease: technical performance derived from OPMDs and OSCC, with implications and limitations for OSF.

Modality	Diagnostic Performance (OPMDs/OSCC)	Resolution	Penetration Depth	Evidence in OSF	Relevance to OSF
Confocal Reflectance Microscopy (CRM)	Sensitivity 80%–90% Specificity 80%–95%	∼1–5 µm (lateral)	200–300 µm	Limited (*ex vivo* OSF only) (75)	Superficial epithelial-stromal assessment only; inadequate for deep fibrosis
Optical Coherence Tomography (OCT)	Sensitivity 75%–90% Specificity 80%–93%	∼10–15 µm (axial), ∼20 µm (lateral)	1–2 mm	Limited (*in vivo* OSF) (76)	Early OSF detection (epithelial thinning); grading and muscle involvement unvalidated
Narrow-Band Imaging (NBI)	Sensitivity 85%–95% Specificity 75%–90%	NA	0.2–0.5 mm	None	Indirect; reflects secondary vascular changes; not fibrosis severity
Raman Spectroscopy (RS)	Sensitivity 85%–98% Specificity 80%–95%	Molecular	300–1,000 µm	Limited (OSF spectra reported, biomarkers unstandardized) (77)	Biochemical plausibility only; OSF-specific thresholds lacking

Reported sensitivity and specificity values are derived predominantly from studies in OPMDs and OSCC. With the exception of limited OCT- and confocal-based investigations (75, 78), these performance metrics have not been validated in OSF.

### 4.1 Confocal reflectance microscopy (CRM)

To Confocal reflectance microscopy (CRM) is a laser-based imaging modality that illuminates targted tissue areas with an 830-nm near-infrared. The reflected and backscattered incident light by subcellular and extracellular microstructures within the tissue generates resultant signal that produces high-resolution, grayscale images. Melanin, keratin, and collagen are highly reflective structures appearing as hyper-reflective (white or bright) elements and contrasting with the surrounding tissue architecture in cutaneous applications. Due to its strong *in vivo* correlation with histopathological findings, CRM has been approved by the U.S. Food and Drug Administration (FDA) as a non-invasive, adjunctive optical imaging tool for the evaluation of skin neoplasms. Two commercially available *in vivo* CRM systems include the wide-probe VivaScope 1500 and the handheld VivaScope 3000 (CaliberID, Rochester, NY). CRM provides lateral (horizon-tal) resolution of 0.5–1 μm and axial resolution of 3–5 μm, with imaging depth ranging from 150 to 200 μm, and a maximum penetration depth of approximately 300 μm. As such, detailed of the full thickness of the epidermis and the underlying papillary dermis can be visualised. Furthermore, integrated software enables automated stitching of sequential optical sections, enhancing the field of view and effectively enabling an “optical biopsy” for broader tissue assessment (79, 80). In the oral cavity, CRM provided detailed, high-resolution, cellular, and subcellular images of normal tongue and lip mucosa. Epithelial cell border and its nuclei in the superficial layer, and extracellular matrix, blood flow, and blood cells in the lamina propria were visible in these images (81). In head and neck tumours, the usefulness of CRM was assessed by capturing images of fresh, unfixed, and unstained surgically excised tumour specimens. Confocal images, thus generated, displayed discernible features of microscopic soft and hard structures. Though not as de-tailed, the images correlated well with histologic findings (82). *In vivo* CRM studies have largely focused on normal oral mucosa and some OPMDs and mucocutaneous disorders (83, 84). Recent investigations utilizing the handheld VivaScope 3000 have demonstrated its applicability in the *in vivo* imaging of both healthy oral mucosa and a range of oral lesions, including white, red, and pigmented types. Confocal images of normal lining mucosa showed a uniform sheet of large epithelial cells with small, round, hyper-reflective nucleoli encircled by hypo reflective perinuclear halos in the superficial epithelial layer. The size of epithelial in the deeper epithelial layers were progressively smaller and more ellipsoidal aand polygonal especially at the epithelial–connective tissue interface. Within the lamina propria of masticatory mucosa, connective tissue fibres appeared as bright, reticulate structures, with a denser and more organized pattern observed in the masticatory mucosa. However, in traumatised mucosa undergoing fibrotic remodelling, the lamina propria exhibited thickened and intensely reflective. Furthermore, confocal imaging revealed disruption of epithelial architecture in leukoplakia and oral squamous cell carcinoma (OSCC). Additionally, aberrant vascular morphologies were consistently observed within the lamina propria in confocal images of OSCC tissues, highlighting the potential of reflectance confocal microscopy for real-time, non-invasive characterization of oral mucosal pathology (85). *In vivo* study of clinically normal tongue mucosa using CRM revealed “dispersed nuclei” in the epithelium with low-density, regularly spaced pattern of epithelial nuclei, a distribution corresponded well with the characteristic cellular organization observed in histological sections of normal oral epithelium. However, the epithelial nuclei of dysplastic epithelium appeared increasingly crowded and densely pack with disruption in its orderly arrangement. The disordered tissue structure was more marked in the confocal images of OSCC, characterized by a loss of architectural definition, making it challenging to distinguish individual cellular elements and other tissue components. These findings underscore the potential of confocal microscopy to detect progressive architectural disorganization associated with malignant transformation in oral mucosa (86).

Further *ex vivo* confocal imaging of normal oral epithelium demonstrated similar distinct nuclear morphologies corresponding to epithelial stratification. Located approximately 50 μm beneath the surface, the large, condensed nuclei, while the intermediate layer, displayed smaller, uniform nuclei were observed in the superficial epithelial layer. Cellular density increased and a higher nuclear-to-cytoplasmic ratio were noted, consistent with normal epithelial maturation patterns in the deeper epithelia layer In contrast, markedly increased nuclear density and speckled structures representing large, homogeneous keratin pearls, which appeared as speckled structures were observed in confocal images of OSCC. Dark and hyper-reflective in the deeper submucosal tissues represented muscle fibres and fibrotic regions respectively. These findings further highlight the pathobiology-driven diagnostic potential of confocal microscopy for distinguishing normal, dysplastic, and malignant oral mucosal tissues based on architectural and cytological features as mentioned earlier (87). In an *ex vivo* study Anuthama et al. obtained confocal images from healthy individuals, betel chewers/smokers (BC/S), and patients with leukoplakia, OSF, and OSCC, and compared these images with conventional light microscopic images. Quantitative assessments of keratin thickness, keratin density, nuclear features of the spinous and basal epithelial layers, and connective tissue density were performed using image analysis software. Densitometric analysis of confocal images of OSF cases in the study delineated fibrotic regions within the connective tissue with a significant increase in fibrosis from normal tissues to those of betel chewers/smokers (BC/S) subjects. Additionally, nuclear density was also significantly higher in BC/S samples compared to normal controls, indicating early subclinical changes associated with areca nut and tobacco exposure. Hence, comparable detailed architectural and cytologic features, these results certainly highlight the utility of CRM as a non-invasive, high-resolution imaging modality with potential for quantitative asassessment in oral mucosal pathology (75). Advances in technology has allowed CRM has, since its first inception, evolved from its awkwardly large system (81) to a miniature objective lens-based system and fibre optic bundle for better access in the oral cavity (86). A handheld, lightweight CRM device has recently been developed and demonstrated robust performance in imaging in the oral cavity, including anatomically challenging sites such as the retromolar trigone. *In vivo* confocal images of normal oral mucosa captured using this device revealed distinct epithelial cell morphology across multiple depths. Particularly in OSF, connective tissue papillae and vascular structures within the lamina propria at depths of approximately 120 μm and 230 μm, respectively were clearly visualised using this device. In addition, the device also exhibited consistent correlates between confocal images of a clinically diagnosed case of leukoplakia with histopathological features of moderate epithelial dysplasia. These results underscore the utility of handheld CRM for non-invasive, high-resolution assessment of epithelial and subepithelial changes in both normal and pathological oral mucosa (88).

Collectively, although these studies support the ability of CRM to visualise epithelial architecture and superficial lamina propria features *in vivo* and *ex vivo* (75, 81–88), OSF-specific evidence remained limited and largely *ex vivo* (75), constrained by the shallow penetration depth of typically <300 μm impacting its ability to assess deeper submucosal fibrosis and muscel involvement that defined advanced OSF. CRM's most plausible role in OSF is quantifying superficial laminaa propria reflectance changes and epithelial atrophy, rather than grading deep fibrotic bands (83).

### 4.2 Optical coherent tomography (OCT)

OCT is a non-invasive OI technique that uses near-infrared (NIR) laser for capturing cross-sectional images of tissue with high spatial resolution. With a depth penetration ranging from 1 to 2 mm, OCT is well-suited for imaging of oral mucosa for the detection of malignant transformation in OPMDs/OSCC, its ability to interrogate the deeper fibrotic bands of OSF remains questionable (89). Its use of non-ionizing radiation of white visible light is used for exploiting the optical properties of tissues (78). OCT is now the gold standard in retinal imaging. Retinal thickness, vessel density, and choroidal changes are several OCT markers used to diagnose a wide range of diseases in ophthalmology, and their application has now been expanded in cardiology, dermatology, and dentistry (90). Early detection of oral cancers and OPMDs, differentiation of normal mucosa from dysplasia/carcinoma, and evaluation of oral submucous fibrosis are among the applications of OCT in dentistry (91). The implementation of optical techniques provides the opportunity to examine the oral mucosa before biopsy (92). Several OCT types are available: 1) Time-Domain OCT (TD-OCT), 2) Spectral-Domain OCT (SD-OCT)/Fourier-Domain OCT, 3) Swept-Source OCT (SS-OCT), 4) Polarization-Sensitive OCT (PS-OCT), 5) Doppler OCT, 6) Full-Field OCT (93).

In 1998, utilizing a fiber-optic Michelson interferometer, TD-OCT successfully delineated the tooth–gingiva interface was distinctly delineated from the underlying enamel and dentin layers, demonstrating the potential of OCT as a more accurate and reproducible alternative to conventional periodontal probing for the assessment and monitoring of periodontal disease. With enhancement to its currently limited imaging depth penetration of approximately 1 mm, this resolution could significantly broaden the scope of OCT applications, specifically in the evaluation of other oral mucosal pathologies such as OSF, where detailed assessment of epithelial and subepithelial changes is essential for early detection and disease staging (94).

In *ex vivo* imaging of 73 patients encompassing 78 clinically suspected oral lesions, SS-OCT images from Hamdoon et al. demonstrated high visualization rates for key anatomical layers: the keratin layer in 94% of cases, the epithelial layer in 93.5%, and the basement membrane in 97%. Notably, the SS-OCT findings showed strong concordance with histopathological outcomes, especially in the assessment of basement membrane integrity and epithelial architecture, underscoring the potential of SS-OCT as a non-invasive diagnostic adjunct for the early detection and classification of oral mucosal pathologies (95).

Recently, *in vivo* SS-OCT images of site-matched healthy tissues and clinically diagnosed OPMDs, in a cohort of 11 patients consistently revealed a hyper-reflective epithelial layer and a hypo-reflective lamina propria in pathological tissues, relative to their healthy counterparts. These reflective patterns corresponded to an in-crease in epithelial thickness and a reduction in lamina propria thickness. The observed epithelial hyper-reflectance was attributed to cellular hyperproliferation, a characteristic feature of dysplastic transformation. Hence, distinct pathological conditions within the oral cavity may, as mentioned earlier, may generate reproducible and diagnostic optical signatures detectable by OCT, highlighting its potential utility as a non-invasive modality for the visualization and monitoring of ultrastructural changes in OPMDs (93, 96). Separately, Lee et al. demonstrated the capability of SS-OCT to detect microscopic alterations in the oral mucosa of patients with OSF before the manifestation of clinically visible surface changes. Vertical (A-mode) and lateral (B-mode) intensity profiles of the epithelial and lamina propria layers in 44 OSF subjects consistently indicated a significant reduction in the epithelial thickness in OSF tissues compared to healthy controls. Notably, 100% sensitivity and specificity for OSF detection was achieved in epithelial thickness of less than 400 μm, with a reduced standard deviation (SD) of lamina propria reflectance intensity observed in OSF cases lacking overt surface abnormalities.This parameter effectively distinguished OSF (characterized by a small lamina propria SD) from both healthy mucosa and lesional tissue (which exhibited larger SD values). These findings suggest that SS-OCT provides a more reliable and objective diagnostic metric than conventional as-assessments such as maximum mouth opening, particularly for early-stage OSF (76). This work supports early OSF detection and superficial stromal change, however, OCT-based grading across established OSF stages or quantification of deep submucosal or muscle fibrosis requires further evaluation. A recent study employed a deep learning-based segmentation tool to quantitatively analyze SS-OCT images obtained from 40 patients. Imaging was performed on both lesional and contralateral clinically normal sites, and seven quantitative parameters were extracted for evaluation. Among these, increased epithelial thickness and loss of epithelial–stromal boundary visualization demonstrated a strong correlation with carcinoma and were effective in distinguishing benign from dysplastic and malignant tissues. Furthermore, the epithelial attenuation coefficient was identified as a significant discriminator, effectively differentiating benign, mild, and moderate dysplasia from severe dysplasia and invasive carcinoma. Here, advanced image analysis techniques potentially enhance the diagnostic utility of SS-OCT for the stratification of oral mucosal lesions (97). *En face* SS-OCT images of site-matched targeted biopsies at a focal depth of 2 mm demonstrated significant improvement in diagnostic accuracy for OPMDs and OSCC with histopathological correlations. SS-OCT images of OSCC consistently revealed loss of epithelial stratification and the presence of characteristic “icicle-like” structures, indicative of invasive carcinoma. At 2-mm imaging depth, SS-OCT was sufficient to visualize critical microstructural alterations in the superficial epithelium, lamina propria, and associated vasculature, highlighting the utility of SS-OCT for real-time, non-invasive assessment of OPMDs/OSCC, and provide a methodological basis for future OSF-specific studies, particular, in studies using site-matched imaging-guided biopsy for correlation with stromal fibrosis depth and architecture (98).

To enable real-time, *in vivo* visualization of collagen fiber architecture analogous to Picrosirius Red staining under polarized light microscopy (PSR-POL), polarization-sensitive optical coherence tomography (PS-OCT) was employed to non-invasively assess collagen organization within the oral cavity. Depth-resolved birefringence imaging successfully demonstrasted color-coded visualization of collagen fibers across various oral mucosal sites. Collagen bundles in labial mucosa appeared compactly and arranged in a tight, grid-like pattern. In ventral surface of the tongue, however, the collagen fibers exhibited strong birefringence in complex, multidirectional orientations. The lingual mucosa showed more variable collagen arrangements. These developments confer high potential to PS-OCT as a valuable adjunct imaging modality for assessing collagen remodeling and structural changes associated with oral health and disease. PS-OCT is therefore an enabling collagen-architecture tool, however, OSF-focus stage discrimination, reproducibility, and correlation with fibrosis depth remains to be elucidated (99).

OCT *in vivo* imaging of tongue squamous cell carcinoma (SCC) in 4-nitroquinoline-1-oxide (4NQO)-induced murine model revealed distinct morphological alterations in intraepithelial papillary capillary loops (IPCLs), transitioning from small, uniform, and tightly coiled structures in normal mucosa to progressively dilated, irregular, and open loops in dysplastic and malignant lesions. Furthermore, the study showed high-resolution detection of IPCLs as small as 10 μm, with imaging depth sufficient to capture clinically relevant superficial mucosal microvascular changes. Although this model reflects neoplasia-associated angiogenic remodelling rather than OSF *per se*, it illustrates how OCT-based microvascular metrics could be explored as indirect correlates of fibrosis-related vascular compromise described in OSF (Section 3.3) (100).

Recent systematic reviews and meta-analyses have demonstrated that OCT exhibited high sensitivity and specificity, along with superior diagnostic accuracy. Furthermore, OCT image analysis using AI/deep-learning based segmentation tool has been associated with enhanced diagnostic performance compared to clinician-based assessments. As such, when augmented by AI, OCT holds significant potential as a valuable adjunct for guiding biopsy, facilitating early detection, and enabling routine screening of high-risk lesions (73, 101, 102). A portable, point-of-care SD-OCT device has recently been developed, and its performance has been validated in a cohort of 347 cases involving OPMDs and malignant lesions across both community and tertiary care settings. OCT-acquired images analyzed using an automated image processing algorithm demonstrated high sensitivity in distinguishing dysplastic (95%) and malignant lesions (93%). Additionally, an artificial neural network (ANN) was trained to identify tissue features indicative of moderate to severe dysplasia. A support vector machine (SVM) model achieved a sensitivity of 83% in delineating moderate to severe dysplasia. These findings substantiate the potential of OCT, especially the portable model, particularly in low-resource settings, for the screening and surveillance of OPMDs, including OSF (37, 103). Importantly, similar portable OCT worksflows should be optimized and validated for OSF, particular in depth-limited assessment of epithelial thinning and superficial stromal remodeling.

### 4.3 Narrow-band imaging (NBI)

NBI is recently highlighted in an umbrella review as a promising adjunctive tool for identifying malignant change of OPMDs (28), especially in visualizing IPCLs of oral mucosa (104). NBI technology narrows the bandwidth of transmitted light into two wavelength bands; one band between 400 and 430 nm (centered at 415 nm) that penetrates the superficial mucosa and highlights submucosal capillaries in brown, second band wavelength between 525 nm and 555 nm and centered at 540 nm penetrates the submucosal layer and prominent vessels are highlighted in cyan (105). Given that NBI primarily highlights superficial vascular patterns, its relevance to OSF will be indirect, reflecting secondary microvascular changes derived from progressive fibrosis, rather than direct measurement of stromal collagen remodeling. In 2005, Muto et al. examined microvascular proliferation (MVP) in 41 superficial mucosal lesions in the oropharynx and hypopharynx using NBI-magnifying endoscopy. The study observed that brownish vascular patterns, not visible under conventional white light, were visualized clearly with NBI. Furthermore, these patterns corresponded histologically to dilated and proliferated microvessels, i.e., angiogenesis, hence establishing NBI as an angiogenesis-targeted diagnostic tool (106). Tanako et al. examined IPCL patterns of magnified NBI images of 10 normal oral mucosa, 8 non-neoplastic oral mucosa lesions, and 23 OSCC. In the study, normal mucosa exhibited IPCL Type I distribution in which IPCLs appeared as regular brown dots or waved lines. IPCL Type II (similar pattern with Type I but larger caliber) and Type III with elongated and dilated IPCLs were observed predominantly in non-neoplastic lesions. Type IV IPCL, which was characterized by larger vessels and destruction due to mucosal erosion, was noted in OSCC. More importantly, dilatation and aggregation of IPCLs, which are presented as brownish areas in magnified images, were detected in all early OSCC cases, indicating a potential role of NBI in the detection of early OSCC (104). Ottaviani et al. evaluated the reliability of NBI in chemically induced oral lesions in a mouse model and 91 patients with histologically-verified premalignant and malignant lesions. Using IPCL classification developed by Takano et al. (104), the study validated the use of NBI in real-time visualization of neo angiogenesis not only clinically but also mechanistically by matching *in vivo* vascular features to microvascular histopathology. It also demonstrated the efficacy of Takano's IPCL classification in predicting lesion grade, enhancing diagnostic confidence during NBI-magnified oral endoscopy (107). The diagnostic accuracy of NBI in detecting early oral malignancy was further validated recently. OPMDs (16 oral lichen planus and 31 leucoplakia) and 13 OSCC cases were examined using COE, white light imaging (WLI), NBI, and the results were benchmarked against incisional biopsy. WLI and NBI demonstrated statistically significant ability in visualizing the extent of OPMDs than CVI alone. In addition, high-grade IPCLs (Type III and IV) were significantly associated with high malignant transformation features such as moderate epithelial dysplasia and proliferative verrucous leukoplakia. Moreover, NBI-assisted IPCL classification demonstrated superior diagnostic accuracy in the prediction of OSCC, 100% sensitivity, 80.9% specificity, and 85.0% accuracy, respectively. As such, NBI is best suited as a tool for detecting dysplasia/early OSCC within the OSF field-cancerization context rather than for grading OSF fibrosis (108).

### 4.4 Raman spectroscopy (RS)

Raman spectroscopy (RS), elastic scattering spectroscopy (ESS), fluorescence spectroscopy (FS), reflectance optical spectroscopy (ROS), and electrical impedance spectroscopy (EIS) are novel techniques that have been investigated vigorously as potential screening modalities for oral mucosal lesions, utilizing diverse biological samples such as tissue biopsies, saliva, serum, and cytological specimens. Among these, RS has emerged as the most promising non-invasive adjunct tool for lesion visualization, targeted biopsy, and monitoring of disease progression (74, 109). RS is a vibrational spectroscopic technique capable of detecting biochemical and molecular variations within tissues. First described by C.V. Raman, the technique is based on the inelastic scattering of monochromatic light, typically from a laser source, by molecular vibrations. Incident photons interacting with intra-molecular bonds undergo energy shifts corresponding to specific vibrational modes, known as Raman scattering. Distinct peaks and bands that reflect the molecular structure and biochemical composition of the tissue in a Raman spectrum constitutes a unique “molecular fingerprint” of the tissue (110). In a clinical study, *in vivo* Raman spectra were acquired from 85 histologically confirmed OSCC and OPMD cases and 72 healthy subjects using a HE-785 Raman spectrometer with a fiber-optic probe. Spectra were collected from the buccal mucosa, lip, and tongue at both tumor and contralateral normal sites. A diagnostic algorithm using principal component–linear discriminant analysis (PC-LDA) with leave-one-out cross-validation (LOOCV) was developed. Site-specific Raman signatures reflected biochemical differences such as lipid-rich in buccal mucosa, protein-dominant in tongue, and mixed in lip. Disease progression was marked by increased protein, DNA, and hem signals and decreased lipid content, enabling differentiation between OPMDs and OSCC. The algorithm showed classification efficiencies of 99%, 70%, 56%, and 72% for healthy, contralateral normal, OPMD, and OSCC tissues, respectively. In a pooled model, it achieved 100% sensitivity and 98% specificity for distinguishing health from diseased tissues. While OSF cases were included, their spectral profiles were not explicitly reported (111). Distinct Raman spectral profiles clearly differentiated cryopreserved, histologically confirmed OSCC and normal oral mucosa (tongue, buccal mucosa, and gingiva). Normal tissues exhibited dominant peaks at 1,004, 1,156, 1,339, 1,450, 1,523, and 1,656 cm^−1^, while tumor spectra featured peaks at 754, 1,064, 1,168, and 1,220 cm^−1^. These OSCC-specific features were linked to elevated proteins, amide I/III vibrations, increased CH₂ bending, and contributions from tryptophan and phenylalanine. A principal component–quadratic discriminant analysis (PC-QDA) model outperformed PC-LDA in accuracy, sensitivity, and specificity, and achieved higher classification efficiency in a patient-wise cross-validation, highlighting RS's potential for intraoperative tumor detection and margin assessment in OSCC (112). Deshmukh et al. examined the depth-related contributions to Raman spectra from normal oral buccal mucosa and their effectiveness in distinguishing normal from malignant tissues. Spectra were collected from both epithelial and connective tissue surfaces of intact biopsies, as well as from histologically separated layers, comprising 234 spectra from normal tissues and 128 from 15 OSCC samples. Supervised and unsupervised analysis revealed significant spectral overlaps between intact surfaces of normal and OSCC spectral, indicating contributions from both superficial and deeper layers, while separated tissues showed distinct spectral clustering. Nontheless, all normal tissue categories (intact and separated epithelium and connective tissue) were distinguishable from OSCC spectra. High accuracy (94%), correctly identifying 65/68 intact epithelium, 50/53 intact connective tissue, 52/54 separated epithelium, and 55/59 separated connective tissue spectra was achieved using supervised LDA classification, confirming that RS of intact mucosa captures diagnostically meaningful molecular information across tissue layers and reliably differentiates malignant from normal tissue, supporting its potential for non-invasive oral cancer detection (113). The feasibility of Shifted-Excitation Raman Difference Spectroscopy (SERDS) was evaluated as a non-invasive, label-free diagnostic modality for the identification of OSCC as an intervention to remove background signals generated by intense tissue autofluorescence captured often in the abovementioned conventional RS. SERDS was employed to suppress background signals by subtracting spectra acquired at two slightly different excitation wavelengths (783 nm and 785 nm), thereby isolating the true Raman signal. SERDS spectral data of 72 spectra obtained from 12 resected OSCC samples, with both malignant and adjacent healthy mucosal regions were processed using Principal Component Analysis (PCA) followed by Linear Discriminant Analysis (LDA). Cross-validation across patient samples was used to evaluate classification performance. The results demonstrated clear spectral differentiation between malignant and non-malignant tissues. OSCC spectra were characterized by elevated features associated with proteins and nucleic acids, whereas normal tissues showed dominant lipid-associated peaks. With a sensitivity of 86.1%, specificity of 94.4%, an overall classification error of 9.7%, and an area under the receiver operating characteristic (ROC) curve (AUC) of 94.5%, SERDS demonstrated its strong suitability for robust clinical translation in real-time tissue diagnostics to objectively and biochemically discriminate OSCC from healthy mucosa (114). Krishna et al. (77) evaluated the feasibility and diagnostic performance of *in vivo* Raman spectroscopy (RS) in 515 sites across 171 patients with OSCC, OSF, and leukoplakia, and 287 sites in 28 healthy volunteers, using a portable fiber-optic RS system (785 nm excitation). Spectral data were analyzed using a probabilistic multivariate model combining Maximum Representation and Discrimination Feature (MRDF) extraction with Sparse Multinomial Logistic Regression (SMLR). Spectral differences were most pronounced between normal tissue and OSCC (96% sensitivity, 99% specificity), with the later showing elevated protein signals and reduced lipid peaks. Although spectral changes in OPMDs were more subtle, RS combined with multivariate modelling still enabled effective discrimination (99% sensitivity, 98% specificity). Specifically, RS of OSF demonstrated reduced lipid band spectra, indicating membrane or fibrosis-associated changes as previously discussed, and elevated protein and nucleic acid features reflecting increased cellular activity and collagen deposition. These changes are consistent with the premalignant nature of OSF and its potential for malignant transformation. In this cohort, OSF spectral exhibited shifts consistent with fibrosis- and cellularity-related biochemical remodeling, including reduced lipid-associated bands and increased protein/nuclei acid features (77). While these findings support biological plausibility for RS in OSF, OSF-specific spectral biomarkers and clinically meaningful thresholds for grading or longitudinal monitoring remain insufficiently standardized. As such, RS should currently be considered as a promising for OSF that requires larger, site-matched, pathology-correlated validation studies before clinical translation.

## 5 Future directions

Given the paucity of OSF-specific validation studies across most optical modalities, future progress will likely arise from two complementary directions: 1) pathology-anchored, extracellular matrix-focused optical biomarkers that quantify fibrosis-related architecture and stiffness, and 2) computational frameworks, in particular AI, that improves objectivity, enable standardized interpretation, and support scalable clinical workflows. The studies highlighted below are therefore discussed as enabling technologies that could inform OSF research and translation, rather than as evidence of established clinical utility in OSF.

The development of advancing technologies and increasing computational prowess has opened up a new molecular nanotechnology chapter in the investigation of OSF pathogenesis. Ghosh et al. introduced MUSItAF (Multiple Signal Classification for Tissue Autofluorescence), a novel, label-free super-resolution imaging strategy to visualize matrix proteins in fixed tissue sections on a nanoscale, focusing especially on pathological fibrosis and epithelial transformation in oral disease. A computational nanoscopic algorithm (MUSICAL), leveraging the autofluorescent properties of structural proteins such as collagen and keratin, was then developed to overcome the resolution limitations of conventional histopathology. A standard Zeiss Axio Observer Z1 wide-field epifluorescence microscope was used to visualize formalin-fixed paraffin-embedded (FFPE) tissue sections from preclinical and clinical specimens. Reconstruction of high-resolution, depth-resolved images by MUSItAF unraveled disease-specific remodeling patterns of the extracellular matrix (ECM) in rat tail collagen, areca nut-induced mouse skin fibrosis, human oral submucous fibrosis (OSF), OSF with dysplasia, and oral squamous cell carcinoma (OSCC). This stain-free approach offered molecular histopathology insight into oral disease by distinctly identifying the density, orientation, and variance of collagen and keratin fibers across disease stages and tissue layers. Quantitative metrics such as layer-specific protein ratios, fiber orientation variance, and collagen bundle density were also derived and validated against histological and immunohistochemical benchmarks. MUSItAF offers a transformative tool for label-free digital pathology and computational ECM phenotyping. It demonstrates its strong potential to support objective fibrosis phenotyping, stage-associated ECM profiling, and computational fibrosis-associated biomarker discovery. As such, MUSItAF is a potential enabling digital platform to objectively phenotype ECM, with potential downstream relevance to OSF staging and risk stratification once linked to clinical outcomes and validated across cohorts (115).

Another recent study has also quickened the potential clinical adoption of real-time, non-invasive diagnosis of OED and OSCC by showing promising results of combining a novel deep learning framework that engages convolutional neural networks (CNNs) and high-resolution *in vivo* confocal microscopy. A cohort of 59 patients with oral mucosal abnormalities was imaged using the InVivage® confocal laser endomicroscopy with topical application of acriflavine and fluorescein. Of the three CNN models developed from PyTorch framework for automated analysis of 9,168 acquired image frames, the quality filtering model, QMR (Quality Micrograph Refiner) model, achieved an accuracy of 89.5%, effectively identifying diagnostically viable images. The acriflavine-based diagnostic triage APMAC model performed well for classifying lichenoid and low-risk lesions (AUC = 0.94 and 0.91, respectively), though it showed limited performance for identifying non-dysplastic and high-risk lesions. Lastly, fluorescein-based FPMAC model demonstrated high diagnostic accuracy across all four categories that consist of no dysplasia, lichenoid, low-risk, and high-risk with AUC values ranging from 0.90 to 0.96. The high-speed processing power of this system, operating at a speed of <0.1 s per image, making it suitable for clinical integration, demonstrating the feasibility of using AI-enhanced confocal microscopy for real-time diagnostic triage and offering a significant advancement in the early detection and monitoring of potentially malignant oral lesions and OSCC while reducing reliance on invasive biopsies. Of note, these AI-confocal frameworks have been developed primarily for OPMDs/OSCC, OSF-specific training sets and pathology-correlated endpoints are necessary before OSF-specific deployment (116).

Yap et al. evaluated the clinical utility of *in vivo* confocal laser endomicroscopy (CLE) as a high-resolution, real-time digital biopsy tool for diagnosing and monitoring in 13 OPMDs and OSCC. Using the InVivage® scanned fibre confocal endomicroscopy with topical fluorescent agents (acriflavine, fluorescein, PARPi-FL), the study captured subcellular-level images from 13 patients with oral lesions. CLE images showed hallmark, histopathologically-correlated dysplastic features, including increased nuclear-to-cytoplasmic ratio, nuclear pleomorphism, and architectural disorganization. MouthMap™, a digitally annotated screening tool, for precise intraoral localization and standardized CLE image interpretation demonstrated its ability to detect early, subclinical changes not evident on COE, supporting its potential as a non-invasive adjunct for targeted biopsy guidance and longitudinal lesion surveillance. These findings establish a framework for broader clinical adoption and future AI-based diagnostic integration of CLE in oral cancer care. For OSF, the potential translational potential lies in adapting these workflows to capture superficial epithelial-stromal features and developing OSF-specific annotation standards that reflect fibrosis-dominant pathology (117). Together, these three studies illustrate a complementary translational pathway, by combining high-resoluation OI, standardized annotation, and AI-assisted interpretation, that could be adapted for OSF, provided OSF-specific datasets and validation are developed (Table 2) (116, 117).

**Table 2:** Enabling optical and computational imaging studies in oral mucosal disease: relevance to OSF as translational frameworks rather than direct clinical evidence.

Aspect	Yap et al. (117)	Ghosh et al. (115)	Ramani et al. (116)
Primary Disease Context	OPMDs/OSCC	OSF, OSF with dysplsia, OSCC (*ex vivo)*	OPMDs/OSCC
Imaging Modality	*In vivo* CLE InVivage® + MouthMap^™^	Label-free computational nanoscopy (MUSI-tAF)	*In vivo* CLE + CNN
Specimen Type	*In vivo* human lesions	FFPE tissue (includes OSF)	*In vivo* human lesions
OSF-specific Data	None	**Yes (ECM architecture in OSF, *ex vivo)***	None
Resolution	Subcellular [same as Ramani et al. (116)]	Nanoscale (sub-diffraction limit, ∼50–100 nm) via computational nanoscopy	Subcellular (lateral 0.55 µm, axial 5.1 µm)
Output Type	Diagnostic image library + framework for intraoral localization and annotation	Quantitative nanostructural ECM profiles and diagnostic imaging without stains	Real-time diagnostic triage tool (<0.1 s/image) for classifying oral lesions
Innovation	Development of MouthMap™, a standardized intraoral annotation and data localization tool	First autofluorescence-based computational histopathology tool enabling label-free, layer-resolved ECM mapping	First demonstration of CNN-powered automated lesion classification from *in vivo* CLE images
Role in OSF	Workflow model only	**Direct ECM phenotyping**	AI framework only
Clinical Readiness for OSF	Not validated	Research-stage	Not validated

Except for the *ex vivo* ECM-focus study by Ghosh et al., these investigations have not provided direct clinical evidence for OI in OSF. They are included to illustrate enabling technologies, such as standardized annotation, ECM-sensitive imaging, and AI-assisted interpretation, that could be adapted for OSF research pending OSF-specific validation.

Bold text in Table 2 reflects OSF-specific areas have been addressed in the study.

A recent study highlights matrix stiffness and the Piezo1–YAP signalling axis as critical drivers of epithelial-mesenchymal transition (EMT) in OSF and promotes malignant progression of the disease. Xu et al. further emphasize how the fibrotic ECM in OSF contributes to epithelial-mesenchymal transition (EMT) and potentially malignant transformation. Using patient tissues, arecoline-induced rat models, and collagen-based *in vitro* systems mimicking ECM stiffness, the group reiterated previously reported microscopic characteristics of OSF, such as increased collagen deposition and reduced vascularization (118). Atomic force microscopy (AFM)-based nanomechanical profiling in the study corroborated with the previous report of heightened tissue stiffness in OSF (77). Furthermore, *in vivo* implantation of stiff collagen matrices under the oral mucosa of rats replicated EMT changes, confirming stiffness alone can drive this transformation via key mediators, Peizo1 and YAP (Yes-associated protein), mechanistically (118). As such, in principle, objective detection and longitudinal quantification of ECM remodeling in betel-quid chewers could inform risk stratification and timing of preventive interventions aimed at mitigating progression to OSCC (118).

None of the adjunctive tools discussed thus far in this narrative review has been recommended as a replacement for the current gold standard—COE and surgical biopsy with histological analysis in diagnosing OPMDs and OSCC due to varied sensitivity and specificity. Importantly, no OI modalities have yet been validated for grading OSF in accordance with established clinical and histopathological staging systems. Even though NBI has demonstrated high levels of sensitivity and specificity and is the only tool recommended to be used strictly as a clinical adjunct (28), interpretation of NBI results still suffers from a lack of objectivity (107). Strong background fluorescence, on the other hand, poses a major challenge to RS as a promising non-invasive diagnostic tool for OPMDs and OSCC. In addition, specialized instruments and expertise are required. As evinced in this section, the future of non-invasive tools in OPMDs, especially OSF and OSCC diagnosis, would hence rely on a combination of various techniques, such as multiscale and multimodal imaging modalities, to overcome the insufficient sensitivity and spatial resolution of conventional imaging methods. Implementation of AI also holds promise in significantly improving the effectiveness of OPMDs and OSCC diagnosis. Moreover, the demand for comprehensible, real-time results by the clinicians during point-of-care diagnostics necessitates the use of a portable handheld device with advanced machine-learning models (74, 119).

## 6 Conclusions

OSF is characterized by progressive fibrotic and epithelial alterations with a known risk of malignant transformation. Optical imaging modalities including confocal reflectance microscopy (CRM), optical coherence tomography (OCT), narrow-band imaging (NBI), and Raman spectroscopy (RS) have demonstrated diagnostic value across OMPDs and OSCC, and provide biologically plausible approaches for interrogating OSF-related microstructural and biochemical remodeling. However, direct validation in OSF remains sparse except for limited OSF-focused studies in OCT- and confocal-based investigations. Most current evidence for OI performance was derived from OMPDs/OSCC, OSF-specific diagnostic accuracy and staging performance remain largely unestablished. Future work needs to prioritize OSF-specific, pathology-correlated validation; standardization of acquisition and reporting; and clinically meaningful endpoints, including alignment with established OSF staging and fibrosis depth. Multimodel strategies into point-of-care assessment integrating ECM-sensitive imaging with AI-assisted interpretation and standardized annotation frameworks may improve objectivity and scalability, supporting eventual transition into point-of-care assessment while complementing, rather than replacing, conventional clinical examination and biopsy.
